# Virtual reality cognitive-behavioural therapy versus cognitive-behavioural therapy for paranoid delusions: a study protocol for a single-blind multi-Centre randomised controlled superiority trial

**DOI:** 10.1186/s12888-021-03473-y

**Published:** 2021-10-11

**Authors:** M. Berkhof, E. C. D. van der Stouwe, B. Lestestuiver, E. van’t Hag, R. van Grunsven, J. de Jager, E. Kooijmans, C. E. R. Zandee, A. B. P. Staring, R. M. C. A. Pot-Kolder, M. Vos, W. Veling

**Affiliations:** 1 University Medical Centre Groningen, University of Groningen, Groningen, the Netherlands; 2 Parnassia Psychiatry Institute, Hague, The Netherlands; 3 Mental Health Service Organization GGZ Noord-Holland-Noord, Heiloo, Heerhugowaard, The Netherlands; 4 Pro Persona, Arnhem, Arnhem, The Netherlands; 5 Flexible Assertive Community Treatment Team, Outpatient Treatment Center, GGZ Delfland, Delft, The Netherlands; 6 First Episode and Early Detection and Intervention Service, Altrecht Psychiatric Institute, Utrecht, The Netherlands; 7 Department of Clinical Psychology, VU University, Amsterdam, The Netherlands

**Keywords:** Psychotic disorder, Paranoia, Delusions, Social anxiety, Virtual reality, Cognitive Behavioural therapy, Social functioning, Cost-effectivity

## Abstract

**Background:**

Seventy per cent of patients with psychotic disorders has paranoid delusions. Paranoid delusions are associated with significant distress, hospital admission and social isolation. Cognitive-behavioural therapy for psychosis (CBTp) is the primary psychological treatment, but the median effect size is only small to medium. Virtual reality (VR) has a great potential to improve the effectiveness of CBTp. In a previous study, we found that VR based CBT (VRcbt) for paranoid delusions is superior to waiting list. As a next step, a direct comparison with CBTp is needed. The present study aims to investigate whether VRcbt is more effective and cost-effective than regular CBTp in treating paranoid delusions and improving daily life social functioning of patients with psychotic disorders.

**Methods:**

A total of 106 patients with DSM-5 diagnosis of psychotic disorder and at least moderate level of paranoid ideations will be recruited for this multicentre randomized controlled trial (RCT). Patients will be randomized to either VRcbt or standard CBTp for paranoid delusions. VRcbt consists of maximum 16 sessions in virtual social situations that trigger paranoid ideations and distress, delivered in an 8–12 week time frame. Standard CBTp also consists of maximum 16 sessions including exposure and behavioural experiments, delivered in an 8–12 week time frame.

The two groups will be compared at baseline, post-treatment and six months follow-up. Primary outcome is the level of paranoid ideations in daily life social situations, measured with ecological momentary assessments (EMA) at semi-random moments ten times a day during seven days, before and after treatment. Every session, participants and therapists will rate the level of paranoid ideation and global clinical impression.

**Discussion:**

Comparison of VRcbt and CBTp will provide information about the relative (cost-) effectiveness of VRcbt for this population. VRcbt may become a preferred psychological treatment for paranoid delusions and social anxiety in patients with psychotic disorder.

**Trial registration:**

Netherlands Trial Register, NL7758. Registered on 23 May 2019.

**Supplementary Information:**

The online version contains supplementary material available at 10.1186/s12888-021-03473-y.

## Background

Seventy per cent of patients with schizophrenia and other psychotic disorders suffer from paranoid delusions, that are characterized by strong suspiciousness with the unfounded belief that other people are trying to harm them [1]. Paranoid delusions are associated with great distress, anxiety, depression, suicidal thoughts, and hospital admission [2, 3]. Furthermore, patients with paranoid delusions often experience problems in social functioning [4]. To avoid perceived threat, i.e., the fear that others may deliberately cause them harm, patients withdraw from social interactions and crowded places. This complicates daily life, as regular activities such as seeing friends, talking to people, shopping, walking on the street or using public transport pose a major challenge. Many patients are socially isolated, have small social networks, and are unemployed [5]. Since paranoid delusions and associated difficulties in social functioning cause a large burden on patients, effective interventions are of great importance. Main treatment options for schizophrenia and other psychotic disorders are antipsychotic medication and psychological treatment. A meta-analysis calculated a small to medium effect size of 0.44 for antipsychotic medication [6]. Many patients discontinue their medication regime due to the serious side effects of antipsychotics. Cognitive-behavioural therapy for psychosis (CBTp) is the main evidence-based psychological treatment for paranoid delusions [7]. CBTp for paranoid delusions aims to challenge delusional beliefs by means of cognitive restructuring and behavioural interventions such as exposure and experiments testing thoughts and beliefs. However, behavioural interventions are often too stressful for patients, as a result of which they avoid those interventions. Moreover, the meticulous preparation required preceding behavioural interventions is time-consuming for therapists, as a result of which this part of CBTp is often not properly performed in clinical practice [8]. Although CBTp is the most effective psychological treatment for paranoid delusions, a recent meta-analysis reported only a small to medium effect size of 0.36 [9]. Nearly half of the patients with schizophrenia and other psychotic disorders do not benefit from current treatments. Therefore, an improvement of treatment is urgently required. Virtual Reality (VR) has a great potential to improve psychological treatment of paranoid delusions. VR is the computer-generated simulation of a three-dimensional environment in which patients can interact with objects and virtual characters in a seemingly real or physical way using special electronic equipment, such as a helmet with a screen inside (head-mounted display, HMD). VR provides a powerful experience that can be used to help patients with paranoid delusions dealing with environments and social situations that make them paranoid or anxious [10]. In a controlled environment with a therapist’s guidance, patients can practice gradually with personalized exposure exercises and behavioural experiments in social situations. VR allows patients to repeatedly experience difficult daily life situations and practice new behaviour with direct feedback from the therapist. Also, patients are aware of the option to withdraw from the VR environments at any time, which makes VR exposure safer and more accessible compared to exposure within CBTp. The interactive nature of VR enables provocation of emotions and responses similar to real environments [11]. To summarize, VR provides an accessible, safe real-world experience in which patients can practice with difficulties in daily life while being coached by a therapist. Previous studies revealed emerging evidence of the potential to treat mental health problems with VR. In the treatment of patients with anxiety, VR has been proven effective and safe [11, 12]. VR is also safe to use in the treatment of patients with a psychotic disorder, and emerging evidence suggests its effectiveness [13]. A pilot study with thirty patients with psychosis reported a large effect size of 1.3 for a short VR based CBT (VRcbt) intervention targeting persecutory delusions, compared to virtual reality exposure [14]. Our group recently conducted the first randomized controlled trial (RCT) of VRcbt with 116 patients and showed that, compared to waiting list, VRcbt is effective for reducing paranoia (d = 1.6) and anxiety (d = 0.7) in patients with schizophrenia and related psychotic disorders [15]. Additionally, significant improvements were established for ideas of persecution, ideas of social reference, and use of safety behaviours. An advantage of VR, highly valued by both patients and therapists, was the possibility to start exposure immediately and successfully.

Moreover, VRcbt has the potential to improve mental health more cost-effectively. A recent meta-analysis of CBTp showed that patients with paranoid delusions are more likely to benefit from treatment when a higher number of CBTp sessions are offered [16], making CBTp time-consuming and expensive. Furthermore, the availability of CBTp is limited for patients with a psychotic disorder. Less than 10% of patients with psychosis are offered CBTp [17, 18]. Meanwhile, first evidence indicates that VRcbt has the potential to achieve positive results in fewer sessions compared to CBTp [14]. In our previous RCT, we aimed to get an impression of the short-term cost-effectiveness of VRcbt for patients with paranoid delusions compared to TAU, from a societal perspective. The VRcbt treatment condition was more expensive than TAU alone, which was to be expected, as VRcbt was added to regular treatment. The incremental cost-effectiveness ratio (ICER) per quality adjusted life year (QALY) gained, however, was within acceptable limits, as was the ICER for other relevant outcomes. At three month follow-up, the VRcbt group had lower health care costs and reduced costs due to productivity loss compared to the TAU alone group. Moreover, there were no psychiatric admission days for the VRcbt group at follow-up. To summarize, these results indicate acceptable cost-effectiveness of VRcbt, even in comparison to a waiting-list control condition without extra costs. In the current study, we take a next step by investigating effectiveness and cost-effectiveness of VRcbt by comparing VRcbt directly to CBTp.

### Objectives

In this RCT, we aim to investigate differences between VRcbt and CBTp in their effect on the level of paranoid delusion in daily life, level of social activities, proportion of time spent in social company, levels of distress, anxiety and depression. In addition, we aim to investigate the differences between groups in the mean number of sessions needed for clinically meaningful improvement of paranoid delusions in daily life. Finally, we aim to assess the differences between health care costs and production losses over the intervention and follow-up period. We hypothesize that VRcbt is more effective and cost-effective than CBTp for treating paranoid delusions and improving daily life social functioning of patients with schizophrenia and related psychotic disorders.

## Methods

This study is funded by the Brain Foundation Netherlands (grant number HA2017.01.04). The study has been approved by the medical ethical board of University Medical Centre Groningen, Groningen (NL66850.042.18), and is conducted in accordance with the Declaration of Helsinki. The study has been registered prospectively in the Netherlands Trial Register, trial number NL7758.

### Participants

Patients who receive treatment in ambulatory mental health care for a psychotic disorder are eligible for the study. Patients are recruited from Dutch and Belgian mental health treatment centres. We recruit participants in the following ways: 1) through advertisement of the study, by distributing posters and flyers at the participating centres, allowing participants to enrol themselves; and 2) through clinicians who inform their patients and refer eligible and interested participants to the study.

### Inclusion criteria

Patients must meet all of the following criteria to be eligible to participate:

- DSM-5 diagnosis of schizophrenia spectrum or other psychotic disorder.
- At least a moderate level of paranoid delusions (Green Paranoid Thoughts Scale [19] > 40).
- Age 18–65 years.

### Exclusion criteria

Patients who meet any of the following criteria will be excluded from participation:

- An estimated IQ below 70;
- Insufficient command of Dutch language.
- Received CBTp for paranoid delusions in the past 12 months.

### Design

**Fig. 1:**
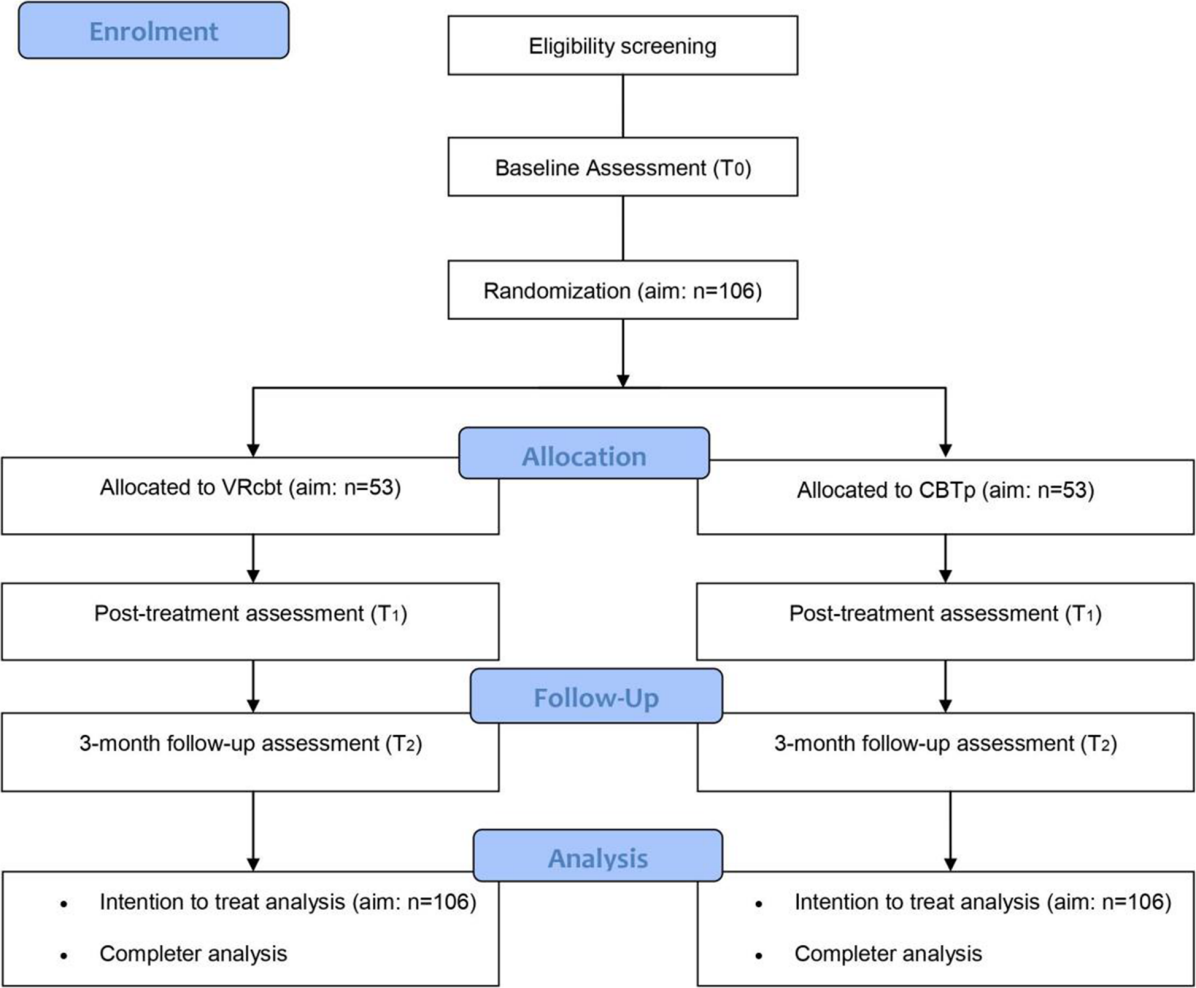
CONSORT flow diagram

This study is a single-blind multicentre randomized controlled trial (RCT) with two conditions: 1) VRcbt for paranoid delusions as experimental condition, and 2) CBTp for paranoid delusions as active control condition. Participants in both conditions may receive other types of treatment as usual, including antipsychotic medication, with the exception of CBT. The effects of the two conditions are compared at baseline (*T*_0_), at post-treatment (*T*_1_), and at three month follow-up (*T*_2_). A Consolidated Standards of Reporting Trials (CONSORT) inclusion flow diagram is shown in Fig. 1.

### Randomization and allocation concealment

Randomization will occur after completion of the baseline assessment. Block randomization will be used, with a block of eight random assignments for each participating mental health centre. The allocator will hide block size from the therapist and research assistants to prevent prediction of the next assignment. After a mental health centre has included eight patients, new blocks will be made available. Allocation to the two conditions will be in a 1:1 ratio. Randomization will be carried out by using the online randomization program www.randomizer.org by an independent researcher of the University Medical Center Groningen who is not involved in the trial. After the baseline assessment, the first author will enter the patients study ID into the online service and receive an email that details the patient’s allocation. The first author will then contact the therapist to inform them of the patients’ allocation. The therapist will then contact the patient about the allocation and to arrange the first therapy session.

### Assessment and blinding

Assessments are carried out by independent research assistants blinded to treatment allocation. This is achieved by instructing coordinators, therapists and participants not to disclose group allocation to research assistants. Research assistants are instructed to stop the assessment in case of unblinding, and another research assistant will repeat the assessment. Other precautions include storing data revealing group allocation (e.g., therapist worksheets) in a separate location and using different assistants for each measurement as much as possible. Blinding is evaluated with a self-report form for research assistants at the end of the post-treatment and follow-up assessments. We will perform a sensitivity analysis by testing the treatment effect only for measurements where research assistants reported being completely blinded to group allocation.

### Power and sample size calculation

In our previous RCT, comparing VRcbt with waiting list, the effect size on Ecological Momentary Assessment (EMA) paranoid delusions (see primary outcome measures) was 1.6 [15]. In the current study, the effect size is likely to be smaller with standard CBTp as active control condition. Assuming a much lower but still clinically relevant effect size of 0.6, a sample size of 122 (allowing 15% drop-out) will have a power of 80% to detect a multically significant treatment effect, using an alpha of 0.05 and a standard deviation of 1.1 for the outcome measure. Taking the multilevel structure of the data into account, with a (conservative) intra correlation coefficient of 0.8 and a commonly applied multiplication factor [20], 106 participants are needed for a unilevel equivalent *N* = 122.

### Interventions

#### VRcbt

VRcbt will be delivered by trained therapists, with at least a postgraduate qualification in CBT, and with a minimum of half a year of experience in psychosis treatment.

VRcbt consists of maximum 16 sessions within an 8–12-week time-frame. Sessions will last maximum 75 min, of which 40 min are spent in virtual social situations that trigger paranoid delusions and distress. The remaining time will be used to plan and reflect on exercises and to complete the session measurements. Throughout the first sessions, therapists construct individually tailored case-formulations based on CBTp, in close collaboration with the patient in order to create a shared understanding of the current paranoid ideas, related feelings and behaviour. Subsequently, patients are guided by therapists who help them drop safety behaviours and test their paranoid beliefs. The following animated virtual social environments can be used, i.e., café, shopping street, supermarket, bus ride, office and living room. The level of difficulty of the particular social environment can be modified by adjusting the number, gender and ethnic appearance of virtual characters (avatars) present in the situation. The level of hostility and suspicious behaviour can be modified as well. Personalized interactive scenarios can be role-played. The therapist talks via a microphone (with voice distortion) as an avatar and operates the avatars’ body movements. Patients wear an Oculus Rift head-mounted display and navigate through the virtual environments using a controller.

#### CBTp

CBTp will be delivered by trained therapists, with at least a postgraduate qualification in CBT, and with a minimum of half a year of experience in psychosis treatment. CBTp also consists of maximum 16 sessions which last up to 75 min within an 8–12 week time-frame. CBTp emphasises cognitive techniques, such as cognitive restructuring and behavioural interventions, including exposure and behavioural experiments. CBTp focuses on exercises aimed at reappraisal of paranoid beliefs’ meaning to reduce distress and improve coping in daily life. Throughout the first sessions, therapists construct individually tailored case-formulations based on CBTp, in close collaboration with the patient in order to create a shared understanding of the current paranoid ideas, related feelings and behaviour. Each session, time will be reserved for planning and reflecting on exercises and completing the session measurements. The Dutch CBT protocol for delusions will be applied (gedachtenuitpluizen.nl).

### Treatment quality and fidelity

Both interventions will be delivered by the same therapists, who are trained in both protocols. All therapists are supervised by a highly skilled and experienced mental health care professional with a registration at the Dutch Association of Behavioural and Cognitive Therapy (VGCt). The VGCt is the scientific association for cognitive-behavioural therapists in the Netherlands. The VGCt is committed to high-quality and scientifically sound development and practice of cognitive-behavioural therapy. For each participant, therapists write a individually tailored case conceptualisation, guided and evaluated after session two by the supervising psychologist. The treatment can only continue after approval of the case conceptualisation. In addition, therapists participate every month in 2-h group supervision sessions, both for VRcbt and CBTp, during which ongoing treatments are presented and discussed. Supervisors meet in online sessions every six weeks. All treatment sessions are audio recorded. A selection of treatment sessions will be rated for treatment fidelity, using the Cognitive Therapy Rating Scale (CTRS) [21]. The CTRS [21] is a reliable [21] and valid [22] instrument to measure treatment fidelity when following a CBTp protocol.

### Materials and measurement instruments

#### Primary outcome measure

##### Momentary paranoia in daily life social situations

Level of momentary paranoia in daily life is measured with *Ecological Momentary Assessment* (EMA). EMA is a structured diary method in which patients are asked to report their momentary thoughts, feelings and symptoms, as well as the (appraisal of the) social context in daily life [23]. Momentary paranoia is measured ten times a day for seven consecutive days before treatment, after treatment and three months after treatment. Items assessing momentary paranoia include “I feel that others might hurt me”, “I feel that others dislike me” and “I feel suspicious” [24]. Items are scored on a 7-point Likert scale ranging from 1 (not at all) to 7 (very). Mean total scores are calculated based on all 70 measurements. The EMA allows investigation of experiences occurring in daily life environments instead of retrospective self-reflection on feelings and behaviour [25]. Therefore, the EMA is less sensitive to recall bias and has a high ecological validity [23].

### Secondary outcome measures

#### Social participation

Social participation is measured by means of EMA. Social participation items assess level of social activities, and proportion of time spent in social company in a natural flow and setting of daily life.

##### Paranoid thoughts

The *Green Paranoid Thoughts Scale* (GPTS) [19] is a self-report questionnaire that consists of two subscales each including sixteen items: part A measures paranoid delusions of social reference and part B measures social persecution, in the past month on a five-point Likert scale. Both scales and their dimensions have good internal consistency and validity [19].

##### Paranoid delusions and hallucinations

The *Psychotic Symptom Rating Scales* (PSYRATS) [26] are semi-structured interviews designed to measure the subjective characteristics of hallucinations and delusions. The PSYRATS has good inter-rater and retest reliability and has good validity, as assessed by internal consistency, sensitivity to change, and in relation to the PANSS [26].

##### Social anxiety

The *Social Interaction Anxiety Scale* (SIAS) [27] consists of nineteen items assessing fear of general social interaction, i.e. distress when meeting and talking with other people, on a five-point Likert scale. High levels of internal consistency and test-retest reliability were established for both scales [27, 28].

##### Depressive symptoms

The *Inventory of Depressive Symptomatology Self-Report* (IDS-SR) [29] is a 30 items self-report questionnaire assessing severity of depressive symptomatology in the past seven days. Psychometric properties of IDS-SR are satisfactory and the instrument has been recommended in research [29, 30].

##### Safety behaviour

The *Safety Behaviours Questionnaire – persecutory delusions* (SBQ) [31] is a semi-structured interview designed to measure safety behaviours, i.e. actions with the aim of reducing persecutory threat. In case a safety behaviour has been reported a patient is asked to rate its frequency over the past month on a four-point scale. The SBQ has a high inter-rater reliability, and an adequate test-retest reliability. The SBQ has adequate validity [31].

##### Worry

The *Penn State Worry Questionnaire* (PSWQ) [32] is a 16-item self-report questionnaire which assesses the trait pathological worry on a five-point scale. The PSWQ has proven to be a reliable and valid measure [32].

##### Self-esteem

The *Self Esteem Rating Scale – Short Form* (SERS-SF) [33], has been designed to measure self-esteem by means of a positive and negative self-esteem subscale. The instrument is a self-report questionnaire that contains 20 items using a seven-point Likert scale. The SERS-SF has shown to be a reliable and valid instrument [33].

##### IPSM

The *Interpersonal Sensitivity Measure* (IPSM) [34] is a self-report questionnaire developed to measure hypersensitivity to interpersonal rejection. The IPSM yields a total score as well as five subscale scores: interpersonal awareness, need for approval, separation anxiety, timidity and fragile inner-self. The IPSM has good psychometric properties [34, 35].

##### Cognitive schemas

The *Brief Core Schema Scales* (BCSS) [36], a 24-item self-report questionnaire, assesses schemata concerning the self and others on four dimensions (negative-self, positive-self, negative-other and positive other) on a five-point Likert scale. The BCSS has good psychometric properties including construct validity [36].

##### Cognitive biases

The *Davos Assessment of Cognitive Biases* (DACOBS) [37] is a self-report questionnaire which assesses cognitive problems and biases on seven independent subscales (jumping to conclusions, belief inflexibility bias, attention for threat bias, external attribution bias, social cognition problems, subjective cognitive problems and safety behaviour) in the past two weeks on a seven-point Likert scale. The DACOBS has proven to be a reliable and valid measure for use in clinical practice and research [37].

#### Cost-effectiveness and cost-utility

##### Healthcare and productivity costs

The *Trimbos Institute and Institute of Medical Technology Assessment questionnaire for Costs associated with Psychiatric illness* (TiC-P) [38] is a self-report questionnaire assessing direct medical costs and productivity costs due to absence from work or reduced efficiency during paid or unpaid costs. The psychometric properties of the TiC-p are satisfactory [38].

##### Quality of life

The *EuroQol Five Dimensions Five Levels* (EQ-5D-5L) [39] is a health related self-report questionnaire which assesses quality of life on five dimensions: mobility, self-care, usual activities, pain/discomfort, and anxiety/depression. Each dimension is rated on a five-level scale that describes the extent of problems in that area. Participants also rate their overall health on the day of the assessment on a 0–100 visual analogue scale (EQ-VAS). The EQ-5D-5L has shown improvement of psychometric properties in relation to the EQ-5D-3 L [40].

##### Clinically meaningful change

In order to test differences between VRcbt and CBT in number of sessions needed to achieve clinically meaningful change, Visual Analogue Scales (VAS), the Sheehan Disability Scale (SDS) [41] and the Clinical Global Impressions Scales (CGI) [42] are used. Patients-ratings on the VAS in combination with SDS will be administered at the beginning of each session, and clinician-ratings on the CGI will be administered at the end of each session. The *Visual Analogue Scales* (VAS) consists of nine items extracted from the GPTS [19], which assesses paranoid thoughts and delusions.

The *Sheehan Disability Scale* (SDS) [41] is a patient-rated measurement on a ten-point scale designed to assess functional impairment in three inter-related domains: work/school, social and family life. The SDS is a psychometrically sound instrument [41]. The *Clinical Global Impression Scale* (CGI) [42] is a brief clinician-rated measurement on a 7-point scale assessing overall symptom severity (i.e. severity of complaints of paranoia safety behaviour and social avoidance) and global improvement (i.e. overall comparison of the patients baseline condition to a ratio of current therapeutically benefit). The CGI applied to schizophrenia has proven to be a valid and reliable instrument to evaluate severity and treatment response [43].

#### Other

##### Presence

The *Igroup Presence Questionnaire* (IPQ) [44] is a fourteen-item self-report questionnaire using a seven-point Likert scale, designed to measure the sense of presence experienced in a virtual environment. The IPQ has established good psychometric properties [44] **Social functioning** The *Personal and Social Performance Scale* (PSP) [45] is an interview designed to assess the extent of disability (1 absent – 5 severe) in four components of social functioning (meaningful activities, personal and social relationships, self-care, and disturbing and aggressive behaviour). The ratings of each component are combined into one score from 0 to 100. The PSP has shown to be a reliable and valid measure [45]. **Demographic background** The questions regarding demographic information include level of education, ethnicity, age, gender, substance use (alcohol, tobacco, cannabis and illicit drugs) and use of medication (antipsychotics, benzodiazepines, other psychotropic drugs).

#### Procedure

Patients who might be eligible for participation will be contacted by their treating clinician and asked if they are interested in participating in the study. Clinicians will ask consent for sharing their contact information with the researchers after which interested patients will be informed about the study and screened by the researchers. After receiving written information about the study, patients will be given a consideration period of one week. If patients decide to participate after one week of consideration, written informed consent will be obtained first, and subsequently patients will complete the GPTS. If the GPTS score is > 40, patients are eligible for the study and the baseline assessment (T_0_) will continue. Following the baseline assessment, the baseline period of EMA will take place. After a week, patients are randomized to either VRcbt or CBTp. Patients allocated to either VRcbt or CBTp, will start with treatment. At the beginning of each session, participants complete the SDS and VAS. At the end of each session therapists complete the CGI. After the treatment period, a post-treatment (T_1_) takes place, followed by a seven days EMA. Finally, a follow-up (T_2_) assessment will be conducted six months after the start of treatment, followed by a final week of EMA. Patients who discontinue participation in the study at an early stage are requested to continue to participate in the measurements.

#### Early completions

In both treatment allocations, early completion will be deliberated when paranoid ideations and avoidance on the CGI scale are rated as zero in two consecutive sessions, and the target behaviour has been achieved. Foregoing applies to all the (virtual) situations formulated in the case formulations in agreement with the supervisor.

#### Data management

Patients data will be coded using a study ID. Personal information and informed consent will be stored separately and safely to ensure privacy. Data will be collected by using an electronic case report form and will be stored in the Research Electronic Data Capture (REDCap) [46]. To evaluate quality and integrity of the research, an independent study monitor will inspect annually.

#### Data analysis

Analysis will be performed according to the intention to treat principle. The groups will be compared at baseline. In case of baseline differences, variables will be added to further analyses as covariates. Potential covariates include age, sex, duration of illness and medication. The effect of VRcbt will be analysed by random intercept mixed effects regression models. The fixed effect of interaction between treatment group (VRcbt or CBTp) and time on momentary paranoia will be fitted as an estimate of the VRcbt treatment effect. Mean scores before and after treatment on each of the dependent variables will be compared between conditions. To determine the number of sessions needed for achieving clinically meaningful change, scores of patients on the VAS, the SDS and the GCI will be compared between conditions. The standard error of measurement (SEM) will be used to determine the proportion of participants with clinically meaningful change in each condition at each time point. Furthermore, cost-effectiveness analyses (CEA) will be conducted using the TiC-P and the EQ-5D-5L questionnaire. The economic evaluation consists of a CEA with improved momentary paranoia in daily life situations and a cost-utility analysis (CUA) with quality-adjusted life years (QALYs) gained as outcome. For both analyses, the incremental cost-effectiveness ratio (ICER) will be calculated as the between-group cost difference divided by the between-group effect difference. The ICER represents the additional costs needed (or saved) for establishing the VRcbt effect. The cost-utility ratio does the same per additional QALY gained. To handle uncertainty in the cost and effect data, nonparametric bootstrapping will be conducted to simulate 2500 ICERs.

## Discussion

In a previous RCT, we compared VRcbt for paranoid delusions to a waiting list [15], as a next step, in this study, we compare VRcbt to CBTp, the latter being the golden standard psychological treatment for psychosis. The main goal of this study is to investigate if the the effect of VRcbt on paranoid delusions in daily life social situations is superior to CBTp. We hypothesize that VRcbt is more (cost-) effective than CBTp for treatment of paranoid delusions and improving daily life social functioning of patients with schizophrenia and related psychotic disorders, and that this difference will be maintained at three month follow-up. This study will add emerging data on VR in the treatment of paranoid delusions. The mean effect size of CBTp for paranoid delusions is only small to medium (d = 0.36) [9], which indicates that treatment needs to be improved. VRcbt has the potential to improve the treatment of paranoid delusions and to be more effective than CBTp, because the risk of avoiding and postponing exposure is much lower. Furthermore, VRcbt enables controllability and environment manipulation, an essential element in treatment of psychosis, but difficult to achieve in a clinical context [11]. Consequently, VRcbt might be more efficient than CBTp as the effect of VRcbt may occur within fewer sessions than CBTp. A systematic review reported a range of 1 to 12 sessions of VR exposure for treatments of anxiety [10], but it is unknown how many VRcbt sessions are needed for treatment of paranoid delusions. In a preliminary pilot study of a short VRcbt intervention for paranoid delusions, reductions in delusional conviction and real-world distress were noted after one session, and a large effect size (d = 1.3) has been reported after six sessions [14]. Our previous study concluded that VRcbt for paranoid delusions is an economically viable approach towards improving patients’ health in a cost-effective manner [47]. Remarkably, while VR is proving its value, studies about the cost-effectiveness of treatment of psychosis and treatment of anxiety in Virtual Reality are limited. Due to emerging technological developments, software and hardware costs have only decreased in recent years. Although VRcbt requires costs in terms of software and hardware, the intervention will be cost-effective when it leads to better outcomes in terms of decreased health care costs (i.e. fewer therapy sessions) and reduced costs due to productivity loss. To conclude, this study will throw light on the (cost)-effectiveness of VRcbt for paranoid delusions. If it proves to be more (cost-) effective than CBTp, VRcbt may become the preferred psychological treatment.

## Supplementary Information

**Additional file 1.**

## Data Availability

Not applicable.

## Abbreviations

BCSS: Brief Core Schema Scales
CBTp: Cognitive-behavioural therapy for psychosis
CGI: Clinical Global Impressions Scales
CTRS: Cognitive Therapy Rating Scale
DACOBS: Davos Assessment of Cognitive Biases
EMA: Ecological Momentary Assessments
EQ-5D-5L: EuroQol Five Dimensions Five Levels
GPTS: Green Paranoid Thoughts Scale
HMD: Head-Mounted Display
ICER: Incremental Cost-Effectiveness Ratio
IDS-SR: Inventory of Depressive Symptomatology Self-Report
IPSM: Interpersonal Sensitivity Measure
IPQ: Igroup Presence Questionnaire
PSP: Personal and Social Performance Scale
PSWQ: Penn State Worry Questionnaire
PSYRATS: Psychotic Symptom Rating Scales
QALY: Quality Adjusted Life Year
RCT: Randomized controlled trial
REDCap: Research Electronic Data Capture
SBQ: Safety Behaviours Questionnaire
SDS: Sheehan Disability Scale
SEM: Standard error of measurement
SERS-SF: Self Esteem Rating Scale – Short Form
SIAS: Social Interaction Anxiety Scale
Study ID: Study Identity
T0: Baseline
T1: Post-treatment
T2: Follow-up
TAU: Treatment As Usual
TiC-P: Trimbos Institute and Institute of Medical Technology Assessment questionnaire for Costs associated with Psychiatric illness
VAS: Visual Analogue Scales
VGCt: Dutch Association of Behavioural and Cognitive Therapy
VR: Virtual Reality
VRcbt: Virtual Reality Cognitive-behavioral therapy

## References

[1] Freeman D, Garety P (2014). Advances in understanding and treating persecutory delusions: a review. Soc Psychiatry Psychiatr Epidemiol.

[2] Freeman D (2016). Persecutory delusions: a cognitive perspective on understanding and treatment. Lancet Psychiatry.

[3] Koyanagi A, Stickley A, Haro JM (2015). Subclinical psychosis and suicidal behavior in England: findings from the 2007 adult psychiatric morbidity survey. Schizophr Res.

[4] Couture SM, Penn DL, Roberts DL (2006). The functional significance of social cognition in schizophrenia: a review. Schizophr Bull.

[5] Gayer-Anderson C, Morgan C (2013). Social networks, support and early psychosis: a systematic review. Epidemiol Psychiatr Sci.

[6] Leucht S, Cipriani A, Spineli L, Mavridis D, Örey D, Richter F, Samara M, Barbui C, Engel RR, Geddes JR, Kissling W, Stapf MP, Lässig B, Salanti G, Davis JM (2013). Comparative efficacy and tolerability of 15 antipsychotic drugs in schizophrenia: a multiple-treatments meta-analysis. Lancet..

[7] Turner DT, Van Der Gaag M, Karyotaki E, Cuijpers P (2014). Psychological interventions for psychosis: a meta-analysis of comparative outcome studies. Am J Psychiatry.

[8] Haddock G, Eisner E, Boone C, Davies G, Coogan C, Barrowclough C (2014). An investigation of the implementation of NICE-recommended CBT interventions for people with schizophrenia. J Ment Health.

[9] Van der Gaag M, Valmaggia LR, Smit F (2014). The effects of individually tailored formulation-based cognitive behavioural therapy in auditory hallucinations and delusions: a meta-analysis. Schizophr Res.

[10] Valmaggia LR, Latif L, Kempton MJ, Rus-Calafell M (2016). Virtual reality in the psychological treatment for mental health problems: an systematic review of recent evidence. Psychiatry Res.

[11] Rus-Calafell M, Garety P, Sason E, Craig TJK, Valmaggia LR (2018). Virtual reality in the assessment and treatment of psychosis: a systematic review of its utility, acceptability and effectiveness. Psychol Med.

[12] Carl E, Stein AT, Levihn-Coon A, Pogue JR, Rothbaum B, Emmelkamp P, Asmundson GJG, Carlbring P, Powers MB (2019). Virtual reality exposure therapy for anxiety and related disorders: a meta-analysis of randomized controlled trials. J Anxiety Disord.

[13] Freeman D, Reeve S, Robinson A, Ehlers A, Clark D, Spanlang B, Slater M (2017). Virtual reality in the assessment, understanding, and treatment of mental health disorders. Psychol Med.

[14] Freeman D, Bradley J, Antley A, Bourke E, DeWeever N, Evans N, Černis E, Sheaves B, Waite F, Dunn G, Slater M, Clark DM (2016). Virtual reality in the treatment of persecutory delusions: randomised controlled experimental study testing how to reduce delusional conviction. Br J Psychiatry.

[15] Pot-Kolder RMCA, Geraets CNW, Veling W, van Beilen M, Staring ABP, Gijsman HJ, Delespaul PAEG, van der Gaag M (2018). Virtual-reality-based cognitive behavioural therapy versus waiting list control for paranoid ideation and social avoidance in patients with psychotic disorders: a single-blind randomised controlled trial. Lancet Psychiatry.

[16] Turner DT, Reijnders M, van der Gaag M, Karyotaki E, Valmaggia LR, Moritz S, Lecomte T, Turkington D, Penadés R, Elkis H, Cather C, Shawyer F, O’Connor K, Li ZJ, de Paiva Barretto EM, Cuijpers P (2020). Efficacy and moderators of cognitive Behavioural therapy for psychosis versus other psychological interventions: an individual-participant data Meta-analysis. Front Psychiatry.

[17] Schizophrenia Commission. The Abandoned Illness. 2012;(November):88.

[18] Staring T, van den Berg D, Schuurmans H, van der Vleugel B. Praten naast pillen: Krijgt de patient met psychose dat wel? 2019:1–20.

[19] Green CEL, Freeman D, Kuipers E, Bebbington P, Fowler D, Dunn G, Garety PA, Measuring ideas of persecution and social reference: the Green (2008). Paranoid Thought Scales (GPTS). Psychol Med.

[20] Stawski RS (2013). Multilevel analysis: an introduction to basic and advanced multilevel modeling (2nd edition). Struct Equ Model A Multidiscip J.

[21] Vallis TM, Shaw BF, Dobson KS (1986). The cognitive therapy scale. Psychometric Properties. J Consult Clin Psychol.

[22] Shaw BF, Elkin I, Yamaguchi J, Olmsted M, Vallis TM, Dobson KS, Lowery A, Sotsky SM, Watkins JT, Imber SD (1999). Therapist competence ratings in relation to clinical outcome in cognitive therapy of depression. J Consult Clin Psychol.

[23] Oorschot M, Lataster T, Thewissen V, Lardinois M, van Os J, Delespaul PAEG, Myin-Germeys I (2012). Symptomatic remission in psychosis and real-life functioning. Br J Psychiatry.

[24] Klippel A, Myin-Germeys I, Chavez-Baldini UY, Preacher KJ, Kempton M, Valmaggia L, Calem M, So S, Beards S, Hubbard K, Gayer-Anderson C, Onyejiaka A, Wichers M, McGuire P, Murray R, Garety P, van Os J, Wykes T, Morgan C, Reininghaus U (2017). Modeling the interplay between psychological processes and adverse, stressful contexts and experiences in pathways to psychosis: an experience sampling study. Schizophr Bull.

[25] Myin-Germeys I, Kasanova Z, Vaessen T, Vachon H, Kirtley O, Viechtbauer W, Reininghaus U (2018). Experience sampling methodology in mental health research: new insights and technical developments. World Psychiatry.

[26] Drake R, Haddock G, Tarrier N, Bentall R, Lewis S (2007). The psychotic symptom rating scales (PSYRATS): their usefulness and properties in first episode psychosis. Schizophr Res.

[27] Mattick RP, Clarke JC (1998). Development and validation of measures of social phobia scrutiny fear and social interaction anxiety11Editor’s note: this article was written before the development of some contemporary measures of social phobia, such as the social phobia and anxiety Inve. Behav Res Ther.

[28] Heidenreich T, Schermelleh-Engel K, Schramm E, Hofmann SG, Stangier U (2011). The factor structure of the social interaction anxiety scale and the social phobia scale. J Anxiety Disord..

[29] Rush AJ, Gullion CM, Basco MR, Jarrett RB, Trivedi MH (1996). The inventory of depressive symptomatology (IDS): psychometric properties. Psychol Med.

[30] Corruble E, Legrand JM, Duret C, Charles G, Guelfi JD (1999). IDS-C and IDS-SR: psychometric properties in depressed in-patients. J Affect Disord.

[31] Freeman D, Garety PA, Kuipers E (2001). Persecutory delusions: developing the understanding of belief maintenance and emotional distress. Psychol Med.

[32] Brown TA, Antony MM, Barlow DH (1992). Psychometric properties of the Penn state worry questionnaire in a clinical anxiety disorders sample. Behav Res Ther.

[33] Lecomte T, Corbière M, Laisné F (2006). Investigating self-esteem in individuals with schizophrenia: relevance of the self-esteem rating scale-short form. Psychiatry Res.

[34] Boyce P, Parker G (1989). Development of a scale to measure interpersonal sensitivity. Aust N Z J Psychiatry.

[35] Harb GC, Heimberg RG, Fresco DM, Schneier FR, Liebowitz MR (2002). The psychometric properties of the interpersonal sensitivity measure in social anxiety disorder. Behav Res Ther.

[36] Fowler D, Freeman D, Smith B (2006). The brief Core Schema scales (BCSS): psychometric properties and associations with paranoia and grandiosity in non-clinical and psychosis samples. Psychol Med.

[37] Van der Gaag M, Schütz C, ten Napel A (2013). Development of the Davos assessment of cognitive biases scale (DACOBS). Schizophr Res.

[38] Bouwmans C, De Jong K, Timman R, et al. Feasibility, reliability and validity of a questionnaire on healthcare consumption and productivity loss in patients with a psychiatric disorder (TiC-P). BMC Health Serv Res. 2013;13:217. 10.1186/1472-6963-13-217 10.1186/1472-6963-13-217 PMC3694473 23768141

[39] Herdman M, Gudex C, Lloyd A, Janssen MF, Kind P, Parkin D, Bonsel G, Badia X (2011). Development and preliminary testing of the new five-level version of EQ-5D (EQ-5D-5L). Qual Life Res.

[40] Janssen MF, Pickard AS, Golicki D, Gudex C, Niewada M, Scalone L, Swinburn P, Busschbach J (2013). Measurement properties of the EQ-5D-5L compared to the EQ-5D-3L across eight patient groups: a multi-country study. Qual Life Res.

[41] Sheehan KH, Sheehan DV (2008). Assessing treatment effects in clinical trials with the Discan metric of the Sheehan disability scale. Int Clin Psychopharmacol.

[42] Guy W. ECDEU assessment manual for psychopharmacology. Dept of Heal Educ Welfare, Public Heal Serv Alcohol, Drug Abus Ment Heal Adm Natl Inst Ment Heal Psychopharmacol Reasearch Branc Devision Extramur Res Programs. 1976.

[43] Haro JM, Kamath SA, Ochoa S, Novick D, Rele K, Fargas A, Rodríguez MJ, Rele R, Orta J, Kharbeng A, Araya S, Gervin M, Alonso J, Mavreas V, Lavrentzou E, Liontos N, Gregor K, Jones PB, on behalf of the SOHO Study Group (2003). The clinical global impression-schizophrenia scale: a simple instrument to measure the diversity of symptoms present in schizophrenia. Acta Psychiatr Scand Suppl.

[44] Schubert T, Friedmann F. The Experience of Presence: Factor Analytic Insights. 2001;10(3):266–81. 10.1162/105474601300343603.

[45] Kawata AK, Revicki DA (2008). Psychometric properties of the personal and social performance scale (PSP) among individuals with schizophrenia living in the community. Qual Life Res.

[46] Harris PA, Taylor R, Thielke R, Payne J, Gonzalez N, Conde JG (2009). Research electronic data capture (REDCap)-a metadata-driven methodology and workflow process for providing translational research informatics support. J Biomed Inform.

[47] Pot-Kolder R, Veling W, Geraets C, Lokkerbol J, Smit F, Jongeneel A, Ising H, van der Gaag M (2020). Cost-effectiveness of virtual reality cognitive behavioral therapy for psychosis: health-economic evaluation within a randomized controlled trial. J Med Internet Res.

