# Supplementary material for: Virtual reality cognitive-behavioural therapy versus cognitive-behavioural therapy for paranoid delusions: a study protocol for a single-blind multi-Centre randomised controlled superiority trial
Source: BMC Psychiatry. 2021 Oct 11;21:496. doi: 10.1186/s12888-021-03473-y (PMC8507393; doi:10.1186/s12888-021-03473-y)
Supplement: Supplementary file 1 — Additional file 1. [file 12888_2021_3473_MOESM1_ESM.docx]

**Additional file**

**Author details**
^1^University Medical Centre Groningen, University of Groningen, the Netherlands, ^2^Parnassia Psychiatry Institute, The Netherlands, ^3^Mental Health Service Organization GGZ Noord-Holland-Noord, Heiloo, The Netherlands, ^4^Pro Persona, Arnhem, The Netherlands, ^5^Flexible Assertive Community Treatment Team, Outpatient Treatment Center, GGZ Delfland, Delft, The Netherlands, ^6^First Episode and Early Detection and Intervention Service, Altrecht Psychiatric Institute, Utrecht, The Netherlands, ^7^Department of Clinical Psychology, VU University, The Netherlands.

**Abbreviations**
BCSS: Brief Core Schema Scales; CBTp: Cognitive-behavioural therapy for psychosis ; CGI: Clinical Global Impressions Scales; CTRS: Cognitive Therapy Rating Scale; DACOBS: Davos Assessment of Cognitive Biases; EMA: Ecological Momentary Assessments; EQ-5D-5L: EuroQol Five Dimensions Five Levels; GPTS: Green Paranoid Thoughts Scale; HMD: Head-Mounted Display; ICER: Incremental Cost-Effectiveness Ratio; IDS-SR: Inventory of Depressive Symptomatology Self-Report; IPSM: Interpersonal Sensitivity Measure; IPQ: Igroup Presence Questionnaire; PSP: Personal and Social Performance Scale; PSWQ: Penn State Worry Questionnaire; PSYRATS: Psychotic Symptom Rating Scales; QALY: Quality Adjusted Life Year; RCT: Randomized controlled trial; REDCap: Research Electronic Data Capture; SBQ: Safety Behaviours Questionnaire; SDS: Sheehan Disability Scale; SEM: Standard error of measurement; SERS-SF: Self Esteem Rating Scale – Short Form; SIAS: Social Interaction Anxiety Scale; Study ID: Study Identity; T0: Baseline; T1: Post-treatment; T2: Follow-up; TAU: Treatment As Usual; TiC-P: Trimbos Institute and Institute of Medical Technology Assessment questionnaire for Costs associated with Psychiatric illness; VAS: Visual Analogue Scales; VGCt: Dutch Association of Behavioural and Cognitive Therapy; VR: Virtual Reality; VRcbt: Virtual Reality Cognitive-behavioral therapy

**Consent for publication**
Not applicable.

**Competing interests**
The authors declare that they have no competing interests.

**Availability of data and materials**
Not applicable.

**Acknowledgements**
We thank the users’ committee for its advisory role. We thank all supervisors, therapists, research assistants and referring clinicians who make this study possible.

**Funding**
This trial is financed by the Brain Foundation Netherlands, grant number HA2017.01.04. The Brain Foundation Netherlands has not played a role in the design of the study, data collection, analysis, and interpretation of data or writing in the manuscript.

**Authors’ contribution**
All authors contributed to the implementation of the study. WV is the principal investigator of the study. MV, AS, and RP-K developed the VRcbt Training Protocol, and MV conducts the training in the VRcbt protocol. MB is responsible for the logistics within the departments of the psychiatric centres: she organizes and supervises the process of patient recruitment, training and managing the research assistants, and monitoring and running the research reports on assessments and diagnosis. CZ, EK, JdJ, MV and RvG supervise therapists individually in case conceptualization and group-wise treatment supervision. MB wrote the first draft of the manuscript, which EvdS and WV critically revised. BL and EvhH helped setting up the trial. All authors have read and approved the final manuscript.

**Ethics approval and consent to participate**
The Medical Ethics Committee has approved the protocol of this study of the University Medical Centre Groningen (METc file number: 2018/ 425, ABR: NL66850.042.18, protocol version 2, 18-04-2019). The study will be conducted in accordance with the principles of the Declaration of Helsinki. Written informed consent to participate in the study will be obtained from all the participants. (Serious) adverse events are recorded and reported to the independent monitor and the Medical Ethical Committee.
